# Comparison of Anatomical and Indication-Based Diagnostic Reference Levels (DRLs) in Head CT Imaging: Implications for Radiation Dose Management

**DOI:** 10.1155/ijbi/6464273

**Published:** 2025-03-19

**Authors:** Benard Ohene-Botwe, Samuel Anim-Sampong, Robert Saizi

**Affiliations:** ^1^Department of Midwifery and Radiography, School of Health & Psychological Sciences, City, University of London, London, UK; ^2^Department of Radiography, University of Ghana, Accra, Ghana; ^3^Department of Radiology, Queen Elizabeth Central Hospital, Blantyre, Malawi

**Keywords:** anatomical, computed tomography, diagnostic reference levels, differences, indication based

## Abstract

**Introduction:** Many diagnostic reference levels (DRLs) in computed tomography (CT) imaging are based mainly on anatomical locations and often overlook variations in radiation exposure due to different clinical indications. While indication-based DRLs, derived from dose descriptors like volume-weighted CT dose index (CTDI_vol_) and dose length product (DLP), are recommended for optimising patient radiation exposure, many studies still use anatomical-based DRL values. This study is aimed at quantifying the differences between anatomical and indication-based DRL values in head CT imaging and assessing its implications for radiation dose management. This will support the narrative when explaining the distinction between indication-based DRLs and anatomical DRLs for patients' dose management.

**Methods:** Employing a retrospective quantitative study design, we developed and compared anatomical and common indication-based DRL values using a dataset of head CT scans with similar characteristics. The indications included in the study were brain tumor/intracranial space-occupying lesion (ISOL), head injury/trauma, stroke, and anatomical examinations. Data analysis was conducted using SPSS Version 29.

**Results:** The findings suggest that using anatomical-based DLP DRL values for CT head examinations leads to underestimations in the median, 25th percentile, and 75th percentile values of head injury/trauma by 20.2%, 30.0%, and 14.5% in single-phase CT head procedures. Conversely, for the entire examination, using anatomical-based DLP DRL as a benchmark for CT stroke DRL overestimates median, 25th percentile, and 75th percentile values by 18.3%, 23.9%, and 13.5%. Brain tumor/ISOL DL*P* values are underestimated by 62.6%, 60.4%, and 71.8%, respectively.

**Conclusion:**The study highlights that using anatomical DLP DRL values for specific indications in head CT scans can lead to underestimated or overestimated DL*P* values, making them less reliable for radiation management compared to indication-based DRLs. Therefore, it is imperative to promote the establishment and use of indication-based DRLs for more accurate dose management in CT imaging.

## 1. Introduction

Computed tomography (CT) imaging can provide detailed images of the internal structures of the body, including bones and soft tissues [1, 2]. The recent advances in image quality, speed, technique robustness, and utility have increased the clinical application of CT technology leading to an increased number of CT examinations performed worldwide [3–6]. Currently, a 4% annual increase has been estimated, resulting in an annual total of approximately 300 million CT scans globally [5].

Despite the benefits of CT and its widespread utilisation, concerns have been raised regarding its impact on health due to the remarkably higher doses of radiation exposure compared to other diagnostic imaging modalities [7]. One of the strategies for enhancing the optimisation of CT radiation doses is the use or application of diagnostic reference level (DRL) [7–10]. According to the International Commission on Radiological Protection (ICRP), a DRL is a form of investigation level used as a tool to support the optimisation of protection in the medical exposure of patients for diagnostic and interventional procedures, and it helps in detecting unusually high radiation doses for common imaging procedures [7]. Quantitatively, a DRL value in CT is described as an arbitrary notional value corresponding to the 75^th^ percentile of dose distribution of the medians of distributions of volume CT dose index (CTDI_vol_) and dose length product (DLP) obtained from surveys of CT examinations [7–11].

The majority of existing DRLs in CT have been established based on anatomical locations such as head, chest, abdomen, and abdomen–pelvis [12–14]. These anatomical DRLs are, however, limited as they do not account for the indications of the procedures. Since CT procedures are based on clinical indications and dictate the use of imaging parameters, indication-based DRLs instead of anatomical DRLs have been strongly recommended by many international bodies [7–11]. The indication-based DRLs are established based on the imaging procedure's clinical indication, taking into consideration patients' clinical histories as well as the reasons for the imaging procedure [6, 15–18]. This has been reported as a valuable optimisation tool for enhancing dose monitoring and optimisation in CT examinations, especially through radiation dose management systems, which provide an additional layer of accountability in the application of ionising radiation [6].

However, it is observed that recent studies [19–26] in CT still generate DRLs based on anatomical parts rather than indication-based DRLs often due to practical limitations and methodological simplicity [7]. This study, therefore, is aimed at quantifying the differences between anatomical and indication-based DRL values in head CT imaging for common indications such as brain lesion/brain tumor/intracranial space-occupying lesion (ISOL), head injury, and stroke. This will help assess its implications for radiation dose management and support the narrative when explaining the distinction between indication-based DRLs and anatomical DRLs for patients' dose management.

## 2. Methods

### 2.1. Ethical Clearance

The Ethics and Protocol Review Committee of the University of Ghana School of Biomedical and Allied Health Sciences (SBAHS/AA/RAD/10997492/2022-2023) and hospital management provided ethical approval and permission to conduct this study. In accordance with the Helsinki protocol, patient privacy, confidentiality, and anonymity were ensured. In particular, patients' identities were assigned to data sets, and names were not recorded. To ensure patients' confidentiality and anonymity, the names on the images were masked and replaced with coded identification numbers. Other details were also anonymised before accessing them.

### 2.2. Study Site

This retrospective cross-sectional, multicentre study was conducted in the CT units of six hospitals in Ghana. These included both private and public hospitals, which have high patient throughput. A summary of the technical specifications of the six CT scanners is presented in Table 1.

### 2.3. Study Data and Sampling

Nonprobability purposive sampling was used to select a population of head CT data sets (images) from adult patients aged 18 years and above who underwent imaging at these study sites between January and June 2023. The head CT scans involved the three most common indications: stroke, head injury/trauma, and brain tumor/ISOL indications. Data sets of all head CT scans together with the detailed radiation dose structured reports were retrieved from the scanners' Picture Archiving and Communication System (PACS) at the study sites.

Information such as patient demographics (age, and gender), technical specifications of CT machines, and acquisition parameters was obtained.

Moreover, dose quantities/descriptors (CTDI_vol_ and DLP) for common head CT indications (stroke, head injury/trauma, and brain lesion/ISOL) and anatomical examinations were obtained from PACS. Scan sequences associated with stroke and head injury/trauma were only in the noncontrast phase, while those for brain lesions/ISOL involved both pre and postcontrast sequences. The contrast and noncontrast protocols at the study site are the same, except for the use of contrast; hence, there are no variations in scanner settings. When collecting the data, scans that were rejected by radiographers or radiologists due to poor image quality were excluded from the study.

From each of the six hospitals, we collected 120 datasets (20 for each indication) to calculate three indication-based DRLs. This resulted in a total of 360 datasets for the study. The anatomical datasets were derived as the median value of the three sets of indication dose data points. This approach was employed because an anatomical DRL is typically developed using CT quantity data or dose descriptors from common head CT examinations, aggregating all indication-specific quantity values for an anatomical part into one. Consequently, 120 anatomical dose datasets were obtained for single-phase (noncontrast) scans and the entire examination (including pre and postcontrast) scans.

To determine the DRLs for the indications in line with the ICRP Publication 135 recommendations [7], the median values for CTDI_vol_ and DLP were first computed for each indication-based examination at each facility. These median values represent the “typical dose” for each respective CT scanner. Subsequently, the 25th and 75th percentiles for CTDI_vol_ and DLP for each indication-based examination and the anatomical indications were calculated. For examinations based on specific indications that required two sequences, like brain tumours/ISOL, DRL values were established for both the single phase and the entire examination. This aligns with the ICRP's recommendation [7] to develop DLP-specific DRLs based on values associated with the entire procedure. To achieve this, we aggregated all total DRLs for various indications and compared them to the total number of exams for each indication. However, CTDI_vol_ was not developed for the entire examination phase as the values are the same as those of single-phase procedures.

Before developing and collecting the dose quantity data, the quality control (QC) test records of the equipment were checked to ensure that the scanners generated the correct data sets. Additionally, all the images associated with quantity data were deemed appropriate by radiologists, as they were reported without any concerns.

### 2.4. Data Analysis

The data were analysed using the Statistical Package for the Social Sciences (SPSS) Version 29 software (Armonk, New York: IBM Corp). Descriptive statistics were computed to summarise the data DRLs and also the comparative analyses between the anatomical and indication based.

## 3. Results

The mean ages of patients who presented with these different indications at the six hospitals ranged from 38.6 ± 14.8 years for head injury/trauma to 59.1 ± 16.8 years for stroke. The pitch, rotation time, and slice thickness used at the various sites for the three conditions were similar. Details of the demographic data and scan parameters are presented in Table 2.

As indicated in Table 3, minor differences in the CTDI_vol_ doses were observed between anatomical and all indications. The differences in anatomical and indication-based DLP DRLs per sequence and in terms of the median, 25th percentile, and 75th percentile are presented in Table 4. It is explained that marginal 25th percentile (3.3%) and 75th percentile (3.1%) differences in the DLP values were observed between anatomical and stroke as well as brain tumor/ISOL indications (25th percentile: 1.9%; 75th percentile: 3.8%). The overall median DLPs for stroke and brain tumor/ISOL indications were lower than the anatomical values for all the CT scanners. The observed differences between anatomical and head injury were large in percentage terms. The variations were 20.2% (overall median), 30.0% (25th percentile), and 14.5% (75th percentile). These differences indicate that higher DLPs were utilised for head injury/trauma protocols compared to anatomical-based DRLs.

The ICRP suggests that DLP-specific DRLs should be developed from the values associated with the entire procedure. The results, as shown in Table 5, explain that for the entire examination, the DRL value differences between anatomical and indication-based 25th percentile DLP DRLs were 23.9% for stroke, 2.3% for head injury/trauma, and 60.4% for brain tumor/ISOL. In terms of the 75th percentiles, the values were 13.5%, 2.3%, and 71.3%, respectively.

**Figure 1:**
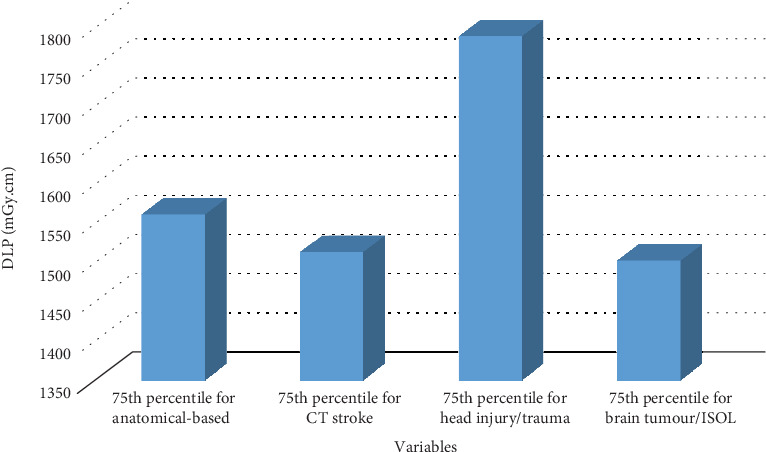
Comparing the 75th percentile value for anatomical-based DRL against indication-based DRL values in terms of DLP for a scan sequence.

**Figure 2:**
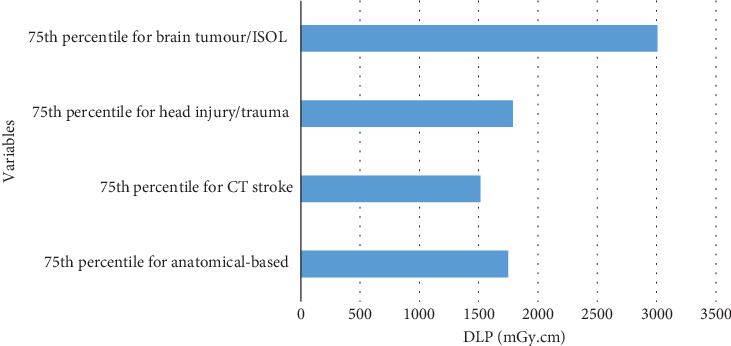
Comparing the 75th percentile value for anatomical-based DRL against indication-based DRL values in terms of DLP for an entire examination.

Figures 1 and 2 compare the 75th percentile values for anatomical-based DRLs against indication-based DRL values in terms of DLP, with Figure 1 focusing on a scan sequence and Figure 2 covering an entire examination.

## 4. Discussions

### 4.1. Scan Parameters

Various combinations of scan parameters were used in undertaking the procedures across the six facilities. Of note, the scan length parameters were specifically adjusted for each common clinical indication when producing images. Notably, the longest scan lengths were utilised for head injury or trauma protocols. In contrast, lower tube current-time products and shorter scan lengths were employed for stroke and brain tumor/ISOL CT scans. This is consistent with expected practice, as head injury protocols typically require imaging of the entire head, from below the chin to the vertex, to adequately visualise both the skull and facial bones, necessitating longer scan lengths. Conversely, scans for conditions such as stroke or cerebrovascular accidents (CVAs) are generally confined to the brain region, resulting in shorter scan lengths and reduced radiation exposure.

It is important to note that the six study sites utilised different types of CT scanners, varying in both make and age. These differences could potentially contribute to variations in dose metrics, including the DRLs. Older CT scanners may lack the advanced dose-optimisation technologies found in newer systems, such as the latest iterative reconstruction algorithms and automatic exposure controls, which can significantly influence dose outputs [3]. Similarly, differences in scanner models and manufacturers may lead to variability in the implementation of dose reduction strategies and image acquisition protocols [6]. Previous studies have highlighted that newer-generation CT scanners tend to achieve lower radiation doses while maintaining diagnostic image quality compared to older systems [7, 8, 15, 27]. This variability underscores the importance of accounting for equipment age and type when interpreting dose data and DRL findings across multiple facilities.

Despite these potential variations, there was a level of standardisation of protocols and practices across the study sites, as they all followed similar standard operating procedures (SOPs). This standardisation is crucial, as differences in imaging protocols can significantly affect dose output [7]. However, it is recognised that flexibility in protocol adjustments is often necessary to accommodate patient-specific factors, such as body habitus, and the clinical task at hand. For instance, the combination of tube current, voltage, and scan length parameters may differ slightly depending on the patient's presentation and the specific diagnostic requirements, leading to minor variations in dose output even with standardised practices.

The DLP, a metric directly correlated with a patient's stochastic risk, increases proportionally with scan length [7, 8, 27].Consequently, any increase or decrease in scan length leads to a corresponding increase or decrease in DLP [15, 28]. This suggests that DRLs may differ between anatomical and indication-based approaches [6–11, 15, 16, 29]. Moreover, differences in scan protocols across centres, despite standardisation efforts, could also influence DRL values. Other authors have reported that variations in scan techniques, including adjustments for patient positioning, exposure settings, and scan range, can contribute to site-specific differences in DRLs [6, 14]. This further emphasises the need for harmonisation of protocols and dose optimisation strategies, particularly when comparing dose data across multiple centres or establishing regional or national DRLs.

### 4.2. Differences Between Anatomical and Indication-Based Dose Outputs for Single Sequences

A single-sequence CT procedure refers to an imaging protocol in which images are acquired using a single scanning phase, whether helical or sequential [7]. The CTDI_vol_, the dose descriptor in this sequence, represents the average radiation dose from a single CT slice adjusted for overlapping slices to estimate the dose delivered to a standard phantom over the scanned volume [28]. The results showed some differences in the median dose quantity values across different scanners and indication procedures. These variations are largely due to the make of the equipment, the combination of exposure factors used, and the patient's head characteristics [2, 29]. However, when aggregated into 75^th^ percentile, for single-sequence procedures, the 25th and 75th percentiles of anatomical CTDI_vol_ values, as well as those based on clinical indications, show minimal variation. These findings suggest that a single anatomical CTDI_vol_ DRL may be suitable for use across multiple clinical indications in head CT imaging.

However, this uniformity does not extend to DRLs that are based on DLP. Specifically, our results show great differences between anatomical and head injury/trauma DLP DRLs. This explains that if an anatomical-based DLP DRL were used to establish DRLs for single-phase head CT scans, rather than indication-based levels, it would underestimate the median, 25th percentile, and 75th percentile values for head injury/trauma by 20.2%, 30.0%, and 14.5%, respectively. These findings support the recommendations by the ICRP that anatomical DRLs could underestimate radiation doses and should be replaced by indication-based DRLs [7].

### 4.3. Differences Between Anatomical and Indication-Based Dose Output for Entire Examination Involving Double Sequences

The ICRP recommends that when establishing DRL values, the DLP values used should be the cumulative DLP for the entire examination, particularly when developing DRLs for multiple scan sequences [7]. Unlike CTDI_vol_, which is the noncumulative dose output value of scans, DLP provides a more comprehensive measure of cumulative patient dose output [28]. Therefore, a DRL value based on the DLP of an entire procedure provides a comprehensive radiation dose benchmark and detailed risk information to enhance patient safety [7, 28]. Despite these guidelines, literature indicates that in many jurisdictions, anatomical DRLs for entire procedures are often used as benchmarks for other procedures involving the same anatomical region rather than specific indications [7].

However, our analysis reveals significant differences in percentage between anatomical DLP DRLs for the entire examination and indication-based DRLs. Specifically, we noted that using an anatomical DRL value instead of indication-based DRLs would overestimate the median, 25th percentile, and 75th percentile values for CT stroke by 18.3%, 23.9%, and 13.5%, respectively. For head injury/trauma, an anatomical DRL would overestimate the median by 13.8% while underestimating the 25th and 75th percentiles by 2.3% and 2%, respectively. Furthermore, employing an anatomical DLP DRL to represent brain tumor/ISOL DLP DRLs, which often involve double sequences, would underestimate the median, 25th percentile, and 75th percentile values by 62.6%, 60.4%, and 71.8%, respectively. Given that DLP is designed to detect unusually high radiation doses for standard imaging procedures [7–11], anatomical-based DLP DRL values are, therefore, not recommended for head CT examinations of specific clinical indications.

A limitation of this study is the lack of consideration of patient-specific parameters such as gender and weight in the analysis. However, for head CT examinations, the anatomical consistency of the region reduces the impact of these variables on DRL values compared to other anatomical regions. Previous studies have suggested that patient weight plays a more significant role in dose variations for body CT scans, where anatomy and body habitus differ more significantly between individuals [14, 16]. While this limitation is unlikely to have affected the findings of this study, future research could incorporate patient-specific parameters to further refine DRL recommendations and understand their potential influence, particularly for other anatomical regions.

## 5. Conclusion

In summary, notable percentage differences in DLP values exist between anatomical and indication-based DRLs in CT examinations. Therefore, using anatomical DLP DRL values for specific indications in head CT scans can result in underestimated or overestimated DLP values, making them less reliable for managing head CT radiation compared to indication-based DRLs. The findings underscore the need to promote the establishment and use of local, national, and regional indication-based DRLs instead of anatomical DRLs, particularly in head CT examinations.

## Figures and Tables

**Table 1:** Technical specifications of CT machines.

**CT ID**	**Manufacturer**	**Model**	**YoM**	**YoI**	**Detector row/slice**
CTS-A	Toshiba	Aquilion One TSX-301A	2012	2012	320/640
CTS-B	GE	Optima 660	2016	2016	64
CTS-C	GE	GE Revolution	2018	2018	64
CTS-D	Siemens	Somatom Emotion	2007	2008	6
CTS-E	Toshiba	Aquilion TSX-101A	2015	2015	16
CTS-F	GE	Lightspeed Pro 16	2011	2011	16

Abbreviations: CT ID, CT identification; CTS, CT scanner; YoI, year of installation; YoM, year of manufacture.

**Table 2:** Demographic data and scanning parameters.

**Demographics**			**Scan parameters (** **m** **e** **a** **n** ± **standard** **deviation)**					
**CT ID**	**Mean age (years)**	**Gender (M/F)**	**Tube voltage (kVp)**	**Tube-current-time product (mAs)**	**Pitch**	**Rotation time (s)**	**Slice thickness (mm)**	**Scan length (mm)**
*Stroke*								
CTS-A	54.1 ± 18.2	6/14	120.0 ± 0.0	225.0 ± 0.0	0.7 ± 0.0	0.8 ± 0.0	5.0 ± 0.0	156.2 ± 11.2
CTS-B	51.3 ± 7.9	10/10	120.0 ± 0.0	180.0 ± 0.0	1.0 ± 0.0	0.6 ± 0.0	5.0 ± 0.0	187.0 ± 12.0
CTS-C	70.3 ± 17.2	11/9	120.0 ± 0.0	129.0 ± 26.5	0.9 ± 0.0	1.0 ± 0.0	5.0 ± 0.0	170.3 ± 18.8
CTS-D	66.5 ± 18.1	11/9	130.0 ± 0.0	250.0 ± 0.0	0.5 ± 0.1	1.0 ± 0.0	3.3 ± 0.9	215.1 ± 14.0
CTS-E	59.0 ± 16.4	12/8	120.0 ± 0.0	225.0 ± 0.0	0.7 ± 0.0	0.7 ± 0.1	5.0 ± 0.0	153.0 ± 15.1
CTS-F	53.8 ± 13.0	13/7	120.0 ± 0.0	225.0 ± 0.0	0.7 ± 0.0	0.8 ± 0.0	5.0 ± 0.0	172.1 ± 7.6
*Head injury/trauma*								
CTS-A	37.5 ± 11.6	12/8	120.0 ± 0.0	225.0 ± 0.0	0.7 ± 0.0	0.8 ± 0.0	5.0 ± 0.0	215.8 ± 35.5
CTS-B	30.4 ± 9.4	15/5	120.0 ± 0.0	180.0 ± 0.0	1.0 ± 0.0	0.6 ± 0.0	5.0 ± 0.0	185.0 ± 18.9
CTS-C	46.6 ± 16.8	10/10	120.0 ± 0.0	190.7 ± 79.2	0.9 ± 0.1	1.5 ± 0.5	5.0 ± 0.0	202.7 ± 34.9
CTS-D	38.0 ± 16.5	16/4	130.0 ± 0.0	250.0 ± 0.0	0.7 ± 0.2	1.0 ± 0.0	3.6 ± 1.2	226.8 ± 30.3
CTS-E	41.3 ± 17.8	16/4	120.0 ± 0.0	225.0 ± 0.0	0.7 ± 0.0	0.8 ± 0.0	5.0 ± 0.0	195.6 ± 25.1
CTS-F	37.6 ± 11.7	5/15	120.0 ± 0.0	225.0 ± 0.0	0.7 ± 0.0	1.05 ± 0.3	5.0 ± 0.0	174.3 ± 22.7
*Brain tumour/ISOL*								
CTS-A	46.9 ± 21.0	11/9	120.0 ± 0.0	225.0 ± 0.0	0.7 ± 0.0	0.8 ± 0.0	5.0 ± 0.0	162.7 ± 17.3
CTS-B	36.8 ± 14.0	12/8	120.0 ± 0.0	180.0 ± 0.0	1.0 ± 0.0	0.6 ± 0.0	5.0 ± 0.0	190.0 ± 15.7
CTS-C	40.6 ± 10.9	11/9	120.0 ± 0.0	136.0 ± 22.8	0.9 ± 0.0	1.0 ± 0.0	5.0 ± 0.0	181.1 ± 22.0
CTS-D	54.8 ± 22.0	11/9	130.0 ± 0.0	250.0 ± 0.0	0.5 ± 0.1	1.0 ± 0.0	3.2 ± 0.7	172.3 ± 18.3
CTS-E	47.9 ± 21.4	11/9	120.0 ± 0.0	225.0 ± 0.0	0.7 ± 0.0	0.8 ± 0.0	5.0 ± 0.0	160.7 ± 29.4
CTS-F	39.9 ± 9.4	8/12	120.0 ± 0.0	225.0 ± 0.0	0.7 ± 0.0	0.8 ± 0.0	5.0 ± 0.0	162.8 ± 29.6

*Note:* Since anatomical DRLs were derived from an aggregate of indication-based scans, the scan parameters above apply to both indication-based and anatomical DRLs.

Abbreviations: F, female; ID, identity; ISOL, intracranial space-occupying lesion; M, male.

**Table 3:** Differences between anatomical and indication-based DRLs for CTDI_vol_ per sequence.

**CT ID**	**Anatomical-based CTDI **_**vol**_ ** DRL (mGy)**	**Indication-based CTDI **_**vol**_ ** DRLs (mGy)**					
		**CT stroke**		**CT head injury/trauma**		**CT brain lesion/ISOL**	
	**Median**	**Median**	**% diff.**	**Median**	**% diff.**	**Median**	**% diff.**
A	86.0	86.0	0.0	86.0	0.0	86.0	0.0
B	32.4	32.4	0.0	32.4	0.0	32.4	0.0
C	30.6	29.9	−2.3	35.7	16.7	30.1	−1.6
D	68.4	68.3	−0.2	68.5	0.2	68.4	0.0
E	77.2	77.2	0.0	77.2	0.0	77.2	0.0
F	77.2	77.2	0.0	77.2	0.0	77.2	0.0
Overall median	72.8	72.8	0.0	72.9	0.1	72.8	0.0
25th percentile	32.0	31.8	−0.6	34.9	9.1	31.9	−0.3
75th percentile	79.4	79.4	0.0	79.4	0.0	79.4	0.0

*Note:* The anatomical DRLs were derived as the median value of the three sets of indication dose data points. This approach was employed because an anatomical DRL is typically developed using CT quantity data or dose descriptors from common head CT examinations, aggregating all indication-specific quantity values for an anatomical part into one. % diff., % difference from anatomical indications.

Abbreviations: ID, identity; ISOL, intracranial space-occupying lesion.

**Table 4:** Differences between anatomical and indication DRLs for DLP per sequence.

**CT ID**	**Anatomical-based DLP DRL (mGy.cm)**	**Indication-based DRLs DLP (mGy.cm)**					
		**CT stroke**		**Head injury/trauma**		**Brain tumour/ISOL**	
	**Median**	**Median**	**% diff.**	**Median**	**% diff.**	**Median**	**% diff.**
A	1527.5	1505.8	−1.4	2129.7	39.4	1484.6	−2.8
B	619.3	605.5	−2.2	583.9	−5.7	645.6	4.3
C	598.9	556.3	−7.1	868.8	45.1	564.1	−5.8
D	1197.5	1162.0	−3.0	1623.0	35.5	1155.0	−3.6
E	1482.3	1443.7	−2.6	1675.2	13.0	1443.7	−2.6
F	1559.5	1540.2	−1.2	1598.0	2.5	1559.5	0.0
Overall median	1339.9	1302.9	−2.8	1610.5	20.2	1299.4	−3.0
25th percentile	613.7	593.2	−3.3	797.6	30.0	625.2	1.9
75th percentile	1562.3	1514.4	−3.1	1788.9	14.5	1503.3	−3.8

*Note:* The anatomical DRLs were derived as the median value of the three sets of indication dose data points. This approach was employed because an anatomical DRL is typically developed using CT quantity data or dose descriptors from common head CT examinations, aggregating all indication-specific quantity values for an anatomical part into one. % diff., % difference from anatomical indications.

Abbreviations: ID, identity; ISOL, intracranial space-occupying lesion.

**Table 5:** Differences between anatomical and indication-based DLP DRLs for the entire examination.

**CT ID**	**Anatomical-based median DRLs for entire exams**	**Indication-based DRLs (total DLP) (mGy.cm)**					
		**CT stroke**		**Head injury/trauma**		**Brain lesion/ISOL**	
		**Median**	**% diff.**	**Median**	**% diff.**	**Median**	**% diff.**
A	2129.7	1505.8	−29.3	2129.7	0.0	2969.2	39.4
B	648.8	605.5	−6.7	583.9	−10.0	1291.3	99.0
C	823.1	556.3	−32.4	868.9	5.6	1128.3	37.1
D	1623.0	1162.0	−28.4	1623.0	0.0	2310.0	42.3
E	1598.0	1443.7	−9.7	1675.2	4.8	2887.4	80.7
F	1598.0	1540.2	−3.6	1132.1	−29.2	3119.0	95.2
Overall median	1598.0	1302.9	−18.5	1377.6	−13.8	2598.7	62.6
25th percentile	779.5	593.2	−23.9	797.6	2.3	1250.5	60.4
75th percentile	1749.7	1514.4	−13.5	1788.8	2.2	3006.7	71.8

*Note:* The anatomical DRLs were derived as the median value of the three sets of indication dose data points. This approach was employed because an anatomical DRL is typically developed using CT quantity data or dose descriptors from common head CT examinations, aggregating all indication-specific quantity values for an anatomical part into one. % diff, % difference from anatomical indications.

Abbreviations: ID, identity; ISOL, intracranial space-occupying lesion.

## Data Availability

The data that support the findings of this study are available from the corresponding author upon reasonable request.
